# Enhancement of Single-Photon Emission Rate from InGaAs/GaAs Quantum-Dot/Nanowire Heterostructure by Wire-Groove Nanocavity

**DOI:** 10.3390/nano9050671

**Published:** 2019-05-01

**Authors:** Wei Wei, Xin Yan, Jie Liu, Bing Shen, Wei Luo, Xiaofeng Ma, Xia Zhang

**Affiliations:** 1 School of Mechanical and Electric Engineering, Guangzhou University, Guangzhou 510006, China; jliu@gzhu.edu.cn (J.L.); luowei.leo@gmail.com (W.L.); 2111707055@e.gzhu.edu.cn (X.M.); 2 Photonics Research Centre, Department of Electronic and Information Engineering, The Hong Kong Polytechnic University, Hung Hom, Kowloon, Hong Kong, China; 3 State Key Laboratory of Information Photonics and Optical Communications, Beijing University of Posts and Telecommunications, Beijing 100876, China; xyan@bupt.edu.cn (X.Y.); xzhang@bupt.edu.cn (X.Z.); 4 4catalyzer Inc., Guilford, CT 06437, USA; shenice.bing@gmail.com

**Keywords:** quantum dot, nanowire, single photon, cavity quantum electrodynamics

## Abstract

Spontaneous emission of luminescent material is strongly dependent on the surrounding electromagnetic environment. To enhance the emission rate of a single-photon emitter, we proposed a wire-groove resonant nanocavity around the single-photon emitter. An InGaAs quantum dot embedded in a GaAs nanowire was employed as a site-control single-photon emitter. The nanoscale cavity built by a wire-groove perpendicular to the quantum dot with an extremely narrow width of 10 nm exhibited an extremely small volume of 10 × 40 × 259 nm^3^. Theoretical analysis showed that the emission rate of the quantum dot was dramatically enhanced by 617x due to the Purcell effect induced by the wire-groove cavity. A fast single-photon emitter with a rate of 50.2 GHz can be obtained that speeds up the data rate of the single-photon emitter. This ultrafast single-photon source would be of great significance in quantum information systems and networks.

## 1. Introduction

The single-photon emitter is one of the key building blocks in quantum communications, quantum computing, and quantum metrology [1,2]. Schemes to realize single-photon emission include atoms in gas phase [3], organic molecules [4], color centers [5], and quantum dots (QDs) [6]. As a typical two-level system, epitaxial semiconductor QDs were shown to emit single photons in 2000 and have emerged as one of the most promising single-photon emitters due to their quasi-atom 0-D dimensions, strong quantum confinement effect, and potential integration with semiconductor optoelectronic devices [7,8,9,10]. Deterministic site-control of QDs is critical for multi-quantum-bit operation in quantum information applications. However, the random spatial positions of strain-driven QDs are barriers for positioning in photonic structures to realize a QD-based single-photon emitter. Combining the site-controlled, one-dimensional morphology of nanowires (NWs) with the discrete energy levels of QDs, quantum-dot-in-nanowires (QDNWs) provide single-photon emission with site/dimension controllability for ideal single-photon emitters in quantum systems [11,12,13,14,15,16,17]. Generally, the intrinsic radiative lifetime of a single-photon emitter placed in an unconstrained dielectric structure is of ~10 ns (corresponding to an emission rate of ~100 MHz) in the visible and near infrared spectral regions [18]. This rate is too long to meet the requirements for high-speed quantum information systems. To control the spontaneous emission rate of a single-photon emitter, cavity quantum electrodynamics (QED) was proposed by Weisskopf and Wigner [19,20]. In cavity QED, optical cavities provide an intriguing way to alter the interaction of light with matter and have been employed in a wide range of fields. The spontaneous emission rate is determined by the density of modes of the electromagnetic field and can be modified by shaping the spatial and spectral redistribution of vacuum fluctuations. This is known as the Purcell effect in the context of cavity QED [21]. The modified emission rate can be quantified by the Purcell factor:

(1) $$F_{p}=\frac{\gamma_{SE}}{\gamma_{0}}=3Q\lambda^{3}/4\pi^{2}V$$

where $\gamma_{0}$ is the free-space spontaneous emission rate and $\gamma_{SE}$ is the modified spontaneous emission rate. This equation reveals that a significant enhancement of emission rate requires an optical resonant cavity confining light down to small dimensions and/or stores light for a long time. Consequently, nonabsorbing all-dielectric photonic crystal cavities with high Q factors (>10^6^) [22,23] and dissipative plasmonic nanocavities of nanoscale volume (far beyond the diffraction-limited volume ${\left(\lambda /2\right)}^{3}$ [24,25]) were proposed to increase the Purcell factor. Due to the extreme light concentration and giant local field enhancement, the limitation of the spontaneous emission rate of plasmonic nanocavities is much larger than that of all-dielectric cavities.

In this study, to shrink the dimensions of the mode volume and enhance the spontaneous emission rate, a perpendicular wire-groove metal nanocavity with an extremely narrow width was proposed. With its efficient light generation and precise positioning, an InGaAs/GaAs QDNW was employed as the single-photon emitter. The wire-groove nanocavity was a Fabry–Pérot cavity. Its three dimensions were below the free-space wavelength. To reveal the mechanism of the enhanced spontaneous emission rate by the nanocavity, the finite elements method (FEM) was used to numerically calculate the cavity properties and investigate the impact of the wire-groove nanocavity on the emitting properties of the QD. With optimized structural parameters of the nanocavity, the resonant wavelength of the nanocavity matched the emitting wavelength of the QD. Consequently, its emission rate was dramatically enhanced via the Purcell effect. Hence, the single-photon emitter in our wire-groove nanocavity would enable quantum information systems working at the speed of tens of gigabits per second.

### Model of Wire-Groove Nanocavity

**Figure 1:**
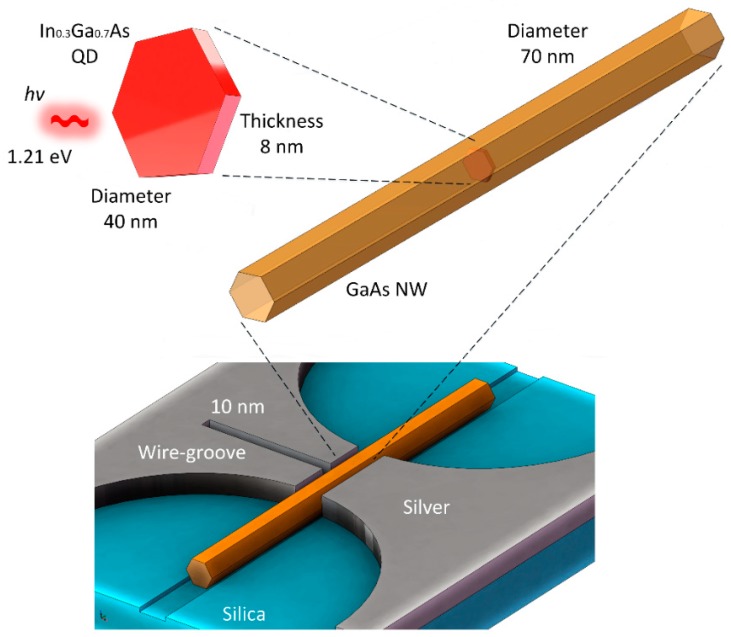
3D schematic diagrams of the wire-groove nanocavity and quantum-dot-in-nanowire (QDNW).

The schematic diagram of the wire-groove nanocavity is shown in Figure 1. On the silica substrate, there was the silver film, for which permittivity is described by the Drude–Lorentz model:

(2) $$\varepsilon \left(\omega \right)=1+\underset{k}{\sum }\frac{\Delta \varepsilon_{k}}{-\omega^{2}-a_{k}\left(i\omega \right)+b_{k}}$$

where $\Delta \varepsilon_{k}$, $a_{k}$, and $b_{k}$ are constants that provide the best fit for silver when compared with optical constant data of silver given by Palik et al. [26]. An InGaAs/GaAs QDNW was placed in the channel of the silver film. The dimensional parameters and indium composition of the QDNW used here were adopted from experimental data in [17] and are marked in Figure 1. With a thickness and diameter of 8 and 40 nm, respectively, the InGaAs QD was embedded in the center of the GaAs NW. The InGaAs QD had a bandgap of 1.21 eV for an indium composition of 0.3. The patterned “bottom-up” approach enabled NW and QD site-control with high surface morphology. The NW can be employed simultaneously as the pump medium to excite the QD and waveguide to guide/collect photons emitted from the QD. Considering the placing deviation of the QDNW, there would be air gaps between the NW surface and channel walls. Thus, the width of the air gap was assumed to be 5 nm here. This narrow low-index air gap induced the hybrid plasmonic modes to confine the optical field at the nanoscale with less-loss propagation [27]. The wire-groove nanocavity in the silver film was perpendicular to the QDNW and connected to the central channel, allowing the coupling of the photons emitted from the QD into the cavity. To enable the nanoscale volume of the cavity, the groove had an extremely narrow width of 10 nm and a thickness equal to the diameter of the QD. It is necessary to note that some values of structural parameters considered in our numerical calculations reached the limit, where the local solutions of macroscopic Maxwell’s equations may not be accurate enough for the description of the electromagnetic properties. For more rigorous investigations, one needs to take nonlocal effects into account [28,29].

## 2. Results and Discussion

**Figure 2:**
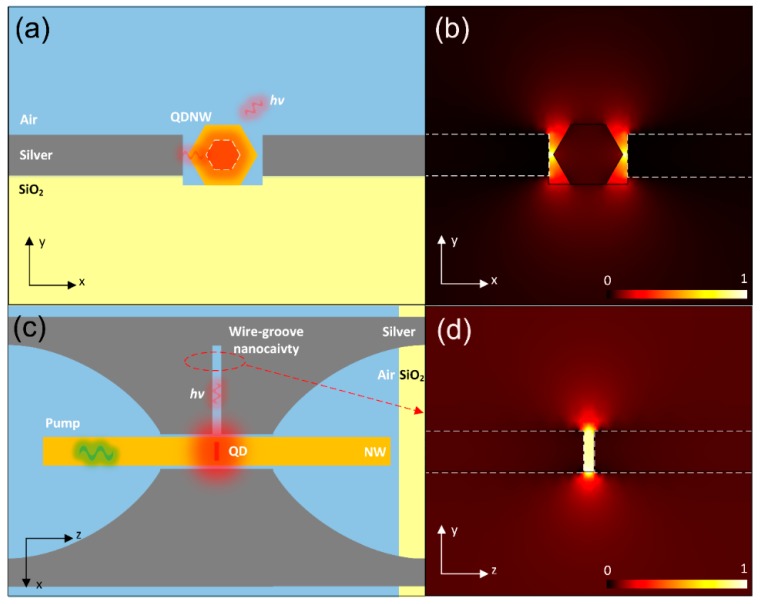
(**a**) Cross-sectional diagram of the QDNW in the channel. (**b**) Corresponding profile of *E*_x_ of the hybrid plasmonic mode. (**c**) Top-view diagram of the wire-groove nanocavity and QDNW. (**d**) Profile of *E*_x_ of the gap plasmon mode in the wire-groove nanocavity.

When the QDNW was placed in the channel, the electromagnetic energy was mainly confined inside the channel, allowing the pumped and emitted photons to propagate into the channel, which is shown in Figure 2. The low-index air gap enabled the hybridization of photonic and plasmonic modes, forming hybrid plasmonic modes. For the x-component of electric field *E*_x_ shown in Figure 2b, the hybrid plasmonic mode was quasi-*TM* in nature, and *E*_x_ concentrated around the air gap between the NW and silver film. Compared with pure surface plasmon modes, the hybrid plasmonic mode was capable of localizing electromagnetic energy at the nanoscale with less metallic dissipation. For the wire-groove shown in Figure 2c,d, the silver nanogroove supported the propagation of electric field *E*_x_ in the form of the gap plasmon mode, exhibiting ultrahigh optical confinement and large effective refractive index [30,31]. Thus, photons emitted from the QD could be coupled into the wire-groove, propagate, and be reflected in the wire-groove nanocavity.

**Figure 3:**
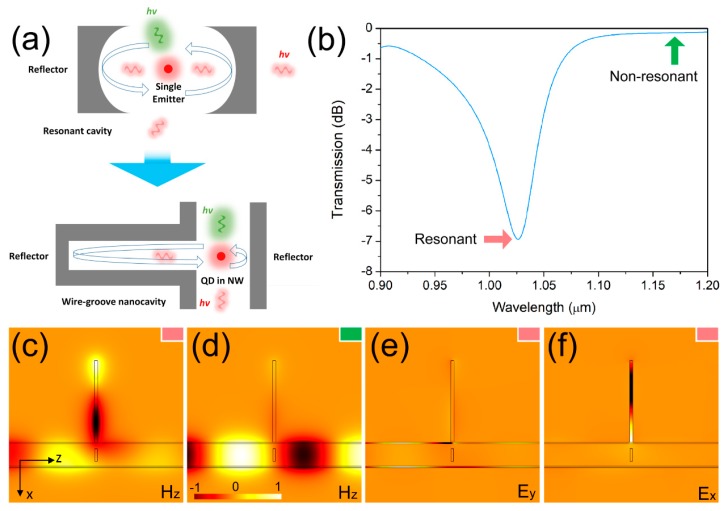
(**a**) Mechanism of the Purcell effect in a micro/nanocavity. (**b**) Transmission spectrum of the nanowire (NW) inside the channel. (**c**) Profile of *H*_z_ of propagating mode at the resonant wavelength. (**d**) Profile of *H*_z_ of propagating mode at the non-resonant wavelength. (**e**) Profile of *E*_y_ of propagating mode at the resonant wavelength. (**f**) Profile of *E*_x_ of propagating mode at the resonant wavelength.

According to Fermi’s golden rule [32], the spontaneous emission rate of an emitter is proportional to the local density of optical states (LDOS). When an emitter is placed in a homogeneous medium, its spontaneous emission rate is constant, depending on the transition dipole moment of the emitter and the permittivity of the surrounding medium. The confinement of the light quanta in the cavity results in the enhancement of the coupling between emitter and field, inducing significant modification of the radiative emitter dynamics. As depicted in the top part of Figure 3a, with single emitter being surrounded by two reflectors in an optical microcavity, the LDOS can be strongly increased along with the spontaneous emission rate. Similar to the above microcavity, the proposed wire-groove nanocavity shown in the bottom part of Figure 3a spatially modified the surrounding dielectric environment of QD and its spontaneous emission rate. For the transmission spectrum of the NW shown in Figure 3b, the resonant wavelength was 1.025 μm, which matched the bandgap of the QD and the photons emitted from the QD couple into the nanocavity and oscillated inside. The optimization of dimensional parameters of the nanocavity to match the bandgap of the QD is elaborated in the next sections. For the profile of *H*_z_ shown in Figure 3c, most electromagnetic energy (around 80% from Figure 3b) coupled into the nanocavity and only some was transmitted through the NW at the resonant wavelength of 1.025 μm. At the non-resonant wavelength (indicated by the green arrow in Figure 3b), most electromagnetic energy was transmitted through the NW without being coupled into the nanocavity (Figure 3d). So, photons emitted from the QD were coupled into the nanocavity, while photons emitted from the NW propagated through the NW without being coupled into the nanocavity. Moreover, the propagation direction in the nanocavity was perpendicular to the propagation direction in the NW. Thus, for the profiles of the electric field shown in Figure 3e,f, when the electromagnetic wave coupled into the nanocavity, the polarized direction of the electric field rotated 90°. So, the polarized direction electric field of the gap plasmon mode inside the nanocavity was perpendicular to that of the hybrid plasmonic mode inside the channel.

**Figure 4:**
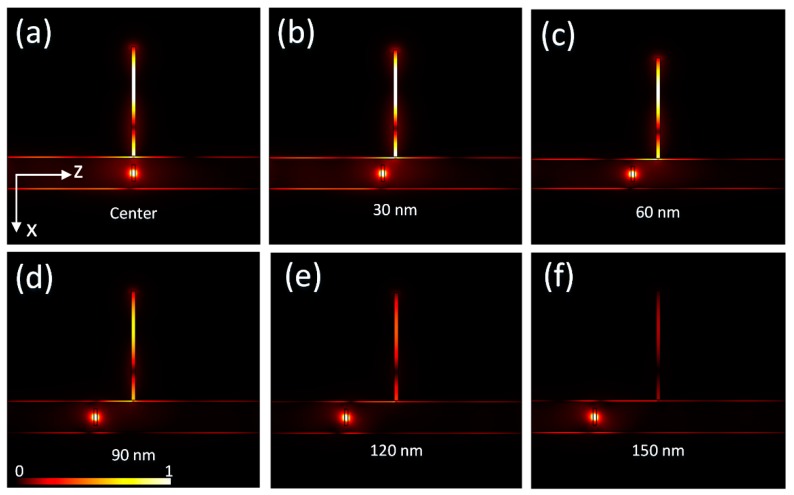
Profiles of the normalized electric field excited by dipole source at the wavelength of 1.025 μm. (**a**–**f**) Position deviation varies from 0 to 150 nm.

Above, we investigated and discussed the wire-groove cavity properties and transmission properties by injecting electromagnetic energy from one end of the NW. To further investigate the coupling between the QD and wire-groove cavity, we needed to directly excite the QD and simulate the electric field profiles. It is well known that the QD is quasi-0-D and can be considered as a dipole source. So, here, a dipole source was employed to mimic the emission of the QD at the wavelength of 1.025 μm. For the normalized electric field profile shown in Figure 4a, the stationary solution showed a strong coupling between the QD and the wire-groove cavity, and the cavity mode overlapped well with the QD emitter. Photons emitted from the QD coupled into the cavity. The displacement deviation of QD had an influence on the coupling strength. As shown in Figure 4b–f, the QD deviated away from the center of the wire-groove cavity and the coupling became weaker with the increasing deviation. When the deviation was below 60 nm, the coupling between the QD and cavity was still strong enough to couple photons into the cavity, which also meant the cavity structure was not sensitive to the displacement deviation of the QDNW. When the deviation was more than 90 nm, the coupling became very weak and it was increasingly difficult for photons to couple into the cavity.

**Figure 5:**
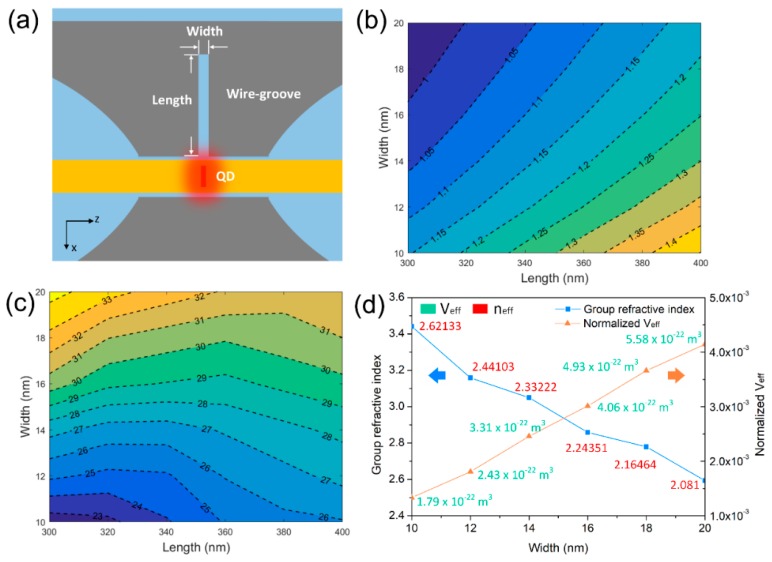
(**a**) Schematic diagram of the nanocavity and QDNW. (**b**) Dependences of the resonant wavelength on nanocavity width and length. (**c**) Dependences of the Q factor on nanocavity width and length. (**d**) Group refractive index and normalized modal effective volume as functions of nanocavity width. Values of $V_{eff}$ and $n_{eff}$ are marked by green or red text, respectively.

Next, we discuss the impact of cavity structures on emission dynamics. The nanocavity length and width are marked and shown in Figure 5a, which both affected the cavity properties. For the dependences of the resonant wavelength $\lambda_{R}$ on the nanocavity width and length depicted in Figure 5b, the resonant wavelength moved towards a longer wavelength with the increasing length. It was consistent with the standing-wave condition in an oscillating cavity:

(3) $$\lambda_{R}\propto 2n_{eff}L$$

where $n_{eff}$ and L are effective refractive index and cavity length, respectively. From the above, the resonant wavelength was directly proportional to the effective refractive index and nanocavity length. As the function between $n_{eff}$ (in *Red*) and width shown in Figure 5d, $n_{eff}$ was inversely proportional to width. The nanocavity length was directly crucial to the resonant wavelength, while the nanocavity width was crucial to the modal effective index $n_{eff}$. Thus, a larger width resulted in a blue shift of the resonant wavelength, while a smaller width resulted in a red shift of the resonant wavelength. Furthermore, by adjusting the parameters of nanocavity width and length, the required resonant wavelength could be obtained to match the tuning composition of the QD single-photon emitter.

The quality factor indicates how long the stored energy remains in the cavity when interband transitions are absent, which is defined as

(4) $$\frac{1}{Q}=\gamma_{cav}/\omega =\frac{\nu_{g,z}\left(\omega \right)}{\omega }\left[\alpha_{i}+\frac{1}{L}\ln\left(\frac{1}{R}\right)\right].$$

This is related to the photon lifetime $\tau_{p}$ entering the rate equation at the resonant frequency ω. A high quality factor indicates a low rate of energy loss relative to the stored energy of the cavity. Further, the oscillations die out slowly and the emission rate is enhanced via the Purcell effect. According to the above equation, both nanocavity length and width have an impact on the Q factor. For the nature of the gap plasmon mode, the modal effective index $n_{eff}$ and group refractive index $n_{g}$ are strongly determined by the gap width. Thus, the group velocity $\nu_{g,z}\left(\omega \right)$ is determined by the nanocavity width, as shown in Figure 5d. The dependences between the Q factor and nanocavity length and width are shown in Figure 5c, in which the higher Q factor located the region of width larger than 17 nm and length smaller than 340 nm. For each length, the Q factor increased with the increasing width. As shown in Figure 5d, the group refractive index $n_{g}$ decreased with the increasing width. Thus, the group velocity $\nu_{g,z}\left(\omega \right)$ increased with the increasing width, resulting in the increasing Q factor. As the nanocavity width increased, the optical field confinement became weaker, which resulted in less metallic dissipation and longer stored energy time in the cavity [33]. The longer nanocavity length also induced the longer stored energy time in the cavity (i.e., the higher Q factor). According to Equation (1), besides the Q factor, the modal effective volume $V_{eff}$ is also crucial to the Purcell factor. Here, to quantify the subwavelength localization scale, the modal effective volume $V_{eff}$ was defined as the product of the modal effective area and nanocavity length. The modal effective area was defined as the ratio of a mode’s total energy density per unit length and its peak energy density [27]:

(5) $$A=\frac{W_{m}}{\max\left\{W\left(r\right)\right\}}=\frac{1}{\max\left\{W\left(r\right)\right\}}\iint_{\infty }W\left(r\right)d^{2}r$$

where W(r) is the energy density for dispersive and lossy materials,

(6) $$W\left(r\right)=\frac{1}{2}\left(\frac{d\left(\varepsilon \left(r\right)\omega \right)}{d\omega }{\left|E\left(r\right)\right|}^{2}+\mu_{0}{\left|H\left(r\right)\right|}^{2}\right).$$

Figure 5d depicts the relationship between the nanocavity width and normalized modal effective volume $V_{n}=V_{eff}/V_{0}$, where $V_{0}={\left(\lambda /2\right)}^{3}$ is the diffraction-limited volume of vacuum. The normalized modal effective volume was directly proportional to the width within a range from 1.3 × 10^−3^ to 4.1 × 10^−3^, which was far beyond the diffraction limit. Such a shrunken modal effective volume would induce a strong Purcell effect and high Purcell factor according to Equation (1), which is elaborated on in the next section.

**Figure 6:**
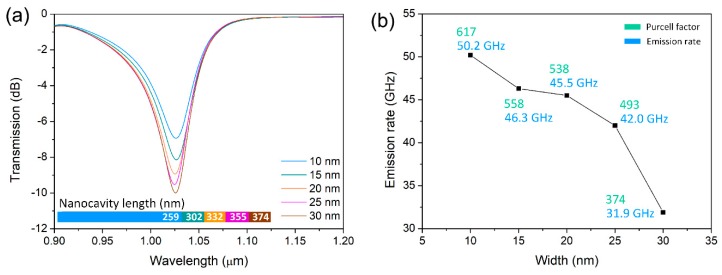
(**a**) Transmission spectra for widths from 10 to 30 nm at the resonant wavelength. (**b**) Emission rate and Purcell factor as functions of nanocavity width.

To match the emission wavelength of the QD single-photon emitter and compare different transmission spectra for varying nanocavity widths, the corresponding nanocavity lengths were optimized by tuning the nanocavity width and length. As the transmission spectra show in Figure 6a, all the central wavelengths were located at 1.025 μm for varying nanocavity widths from 10 to 30 nm. The corresponding nanocavity lengths are marked at the bottom of the figure and have the same trend as that shown in Figure 5b. The coupling strength between the nanocavity and QDNW became more intense with the increasing nanocavity length. This is attributed to the wider nanocavity allowing more electromagnetic energy to be coupled into the cavity. According to the functions of the Purcell factor (in *Green*) and nanocavity width depicted in Figure 6b, the Purcell factor decreased with the increasing width. A maximum Purcell factor of 617 was obtained when the width was 10 nm. According to Equation (1), the Purcell factor is determined by the Q factor and the modal effective volume. However, the impacts of Q factor and modal volume are contradictory. It is known that plasmonic cavities have relatively high loss and very small size. It was difficult to get high Q factors for plasmonic cavities (the highest calculated Q factor was just 34, as shown in Figure 5c). However, the modal effective volume could be efficiently decreased to a scale beyond the diffraction limit. So, at this time, modal effective volume dominated in plasmonic cavities. Although the Q factor was relatively small, the Purcell factor could still be very high due to the nanoscale structures and very small modal effective volume.

A single-photon emitter takes advantage of spontaneous emission from the QD, which depends on the local electromagnetic environment. The modified single-photon emission rate by the wire-groove nanocavity can be described by [18]:

(7) $$\gamma_{SE}=F_{p}\gamma_{0}\exp\left(-L/L_{SPP}\right)$$

where L and $L_{SPP}$ are the nanocavity length and propagation length of the gap plasmon mode inside the nanocavity, respectively. $\gamma_{0}$ is the intrinsic emission rate of the QD emitter, which has a negative correlation with its intrinsic radiative lifetime $\tau_{p}$ of $\gamma_{0}\propto 1/\tau_{p}$. According to the experimental data from [14], the intrinsic radiative lifetime of an In_0.3_Ga_0.7_As/GaAs QDNW is approximately 10 ns. As the single-photon emission rate (in *Blue*) shows in Figure 6b, the emission rate decreased with the increasing width. This is consistent with the above equation that $\gamma_{SE}$ is proportional to the Purcell factor. The proposed enhanced single-photon emitter had a maximum emission rate of 50.2 GHz for a nanocavity width and length of 10 and 259 nm. This rate was more than two orders of magnitude higher than traditional waveguide-based devices [10]. Hence, the single-photon emission rate can be greatly enhanced by the shrinking nanocavity via the Purcell effect.

## 3. Conclusions

In summary, we proposed a fast InGaAs/GaAs QDNW single-photon emitter, for which the spontaneous emission rate was enhanced by a wire-groove nanocavity. The QDNW offered site controllability for the QD single-photon emitter. With an extremely narrow nanocavity width of 10 nm, the electromagnetic field could be greatly squeezed. The corresponding nanocavity length was optimized to be 259 nm to make the cavity resonant wavelength match the emitting wavelength of the QD emitter. Hence, the modal effective volume was far beyond the diffraction limit and significantly modified the electromagnetic environment around the QD. Due to the Purcell effect arising from the modified electromagnetic environment, the spontaneous emission rate was significantly enhanced to a maximum rate of 50.2 GHz. Thus, the wire-groove-nanocavity-based QDNW with a high-speed emission rate is promising for ultrafast single-photon emitters and high-data-rate quantum applications.
